# Efficacy of using tidal volume challenge to improve the reliability of pulse pressure variation reduced in low tidal volume ventilated critically ill patients with decreased respiratory system compliance

**DOI:** 10.1186/s12871-022-01676-8

**Published:** 2022-05-04

**Authors:** Yujun Xu, Jun Guo, Qin Wu, Junjun Chen

**Affiliations:** Department of Critical Care Medicine, West China Hospital, Sichuan University, Chengdu, China

**Keywords:** Pulse pressure variation (PPV), Critically ill, Fluid responsiveness, Tidal volume challenge, Mechanical ventilation, Low tidal volume

## Abstract

**Background:**

The prediction accuracy of pulse pressure variation (PPV) for fluid responsiveness was proposed to be unreliable in low tidal volume (Vt) ventilation. It was suggested that changes in PPV obtained by transiently increasing Vt to 8 ml/kg accurately predicted fluid responsiveness even in subjects receiving low Vt. We assessed whether the changes in PPV induced by a Vt challenge predicted fluid responsiveness in our critically ill subjects ventilated with low Vt 6 ml/kg.

**Methods:**

This study is a prospective single-center study. PPV and other parameters were measured at a Vt of 6 mL/kg, 8 mL/kg, and after volume expansion. The prediction accuracy of PPV and other parameters for fluid responsiveness before and after tidal volume challenge was also analyzed using receiver operating characteristic (ROC) curves.

**Results:**

Thirty-one of the 76 subjects enrolled in the study were responders (41%). Respiratory system compliance of all subjects decreased significantly (26 ± 4.3). The PPV values were significantly higher in the responder group than the non-responder group before (8.8 ± 2.7 vs 6.8 ± 3.1) or after (13.0 ± 1.7 vs 8.5 ± 3.0) Vt challenge. In the receiver operating characteristic curve (ROC) analysis, PPV_6_ showed unsatisfactory predictive capability with an area under the curve (AUC) of 0.69 (95%CI, 0.57–0.79, *p* = 0.002) at a Vt of 6 mL/kg. PPV_8_ andΔPPV_6–8_ showed good predictive capability with an AUC of 0.90 (95% CI, 0.81–0.96, *p* < 0.001) and 0.90 (95% CI, 0.80–0.95, *P* < 0.001) respectively. The corresponding cutoff values were 11% for PPV_8_ and 2% for ΔPPV_6–8_.

**Conclusions:**

PPV shows a poor operative performance as a predictor of fluid responsiveness in critically ill subjects ventilated with a tidal volume of 6 mL/ kg. Vt challenge could improve the predictive accuracy of PPV to a good but not excellent extent when respiratory system compliance decreased significantly.

**Supplementary Information:**

The online version contains supplementary material available at 10.1186/s12871-022-01676-8.

## Quick look

### Current knowledge

Pulse pressure variation (PPV) has been used to predict preload fluid responsiveness in mechanically ventilated subjects. PPV interpretation is doubtful during low tidal volume ventilation (tidal volume ≦ 6 ml/kg), which is increasingly used in ICU subjects. Some studies suggested temporary tidal volume (Vt) challenge could improve the prediction accuracy of PPV. However, reports regarding the effect of the Vt challenge were conflicting.

What this paper contributes to our knowledge.

PPV shows a poor operative performance as a predictor of fluid responsiveness in critical care subjects ventilated with a tidal volume of 6 mL/ kg. Vt challenge could improve the predictive accuracy of PPV to a good but not excellent extent in the context of lung protective ventilation when respiratory system compliance decreased significantly.

## Background

Fluid therapy is the primary resuscitation maneuver of acute circulatory failure management in critically ill subjects [1]. Both under and over-fluid resuscitation with fluid may cause a poor clinical outcome [2]. Testing for fluid responsiveness may help one decide to administer fluid or to stop fluid administration. It is very common that “fluid responsiveness” does not occur in critically ill subjects, who are more vulnerable to volume expansion [3]. Without testing for an individual's fluid responsiveness, volume expansion can lead to increased cardiac filling pressure and fluid overload but not a significant hemodynamic improvement [4]. Clinically, many monitoring indices are implemented to help physicians assess fluid responsiveness [5]. Among these indicators, pulse pressure variation (PPV) has been applied to predict preload fluid responsiveness in mechanically ventilated subjects [6]. Compare to other traditional indicators, the PPV is a dynamic parameter that can be quickly recorded from a bedside monitor and reliably predicts preload responsiveness [7].

Nevertheless, PPV interpretation is doubtful during low tidal volume ventilation (tidal volume ≦ 6 ml/kg), which is increasingly used in ICU subjects, especially those with sepsis and acute respiratory distress syndrome [8, 9]. Some studies attempted to overcome this limitation, one of which suggested that the temporary tidal volume (Vt) challenge could improve the prediction accuracy of PPV [10]. However, reports regarding the effect of the Vt challenge were conflicting, which was not surprising had regard to the difference in population heterogeneity and other settings between studies [11–13].

To evaluate the validity of the Vt challenge, we performed a study on septic shock patients with or without ARDS who were receiving low tidal mechanical ventilation. We also compared the ability of PPV to predict fluid responsiveness (before and after Vt challenge) with additional parameters.

## Materials and methods

Subjects with septic shock who received low tidal mechanical ventilation between October 2017 to May 2020 in the Department of Critical Care Medicine at West China Hospital were screened in the study. The study protocol was reviewed and approved by the Ethics Committee of West China Hospital of Sichuan University (No. 2018–88), and written informed consent was obtained from the subjects' guardians or next of kin.

We included subjects 18 years old or older fulfilling the criteria for a diagnosis of septic shock with or without ARDS, who were receiving low tidal volume ventilation using volume control ventilation and having continuous cardiac output monitoring for whom the treating physician planned to give a fluid bolus. Septic shock was defined according to the Sepsis-3 consensus of the Society of Critical Care Medicine and the European Society of Intensive Care Medicine [14]: vasopressor requirement and serum lactate > 2 mmol/L in the absence of hypovolemia in a patient with suspected or proven infection. For diagnosis of ARDS, the patient must have new or worsening symptoms within 1 week of a known clinical insult; bilateral opacities observable on anteroposterior chest radiographs that were not due to effusions, nodules or lobar or lung collapse; and hypoxemia, defined by a PaO_2_/FiO_2_ < 300 mm Hg and a minimum positive end-expiratory pressure ≥ 5 cm H_2_O, that was not fully explained by cardiac failure or fluid overload [15]. Subjects with cardiac arrhythmias, valvular heart disease, right ventricular dysfunction, intracardiac shunt, air leakage through chest drains, abdominal compartment syndrome, pregnancy, or urgently requiring a fluid bolus were excluded. We excluded patients with right ventricular dysfunction or intracardiac shunt as it has been suggested that they could result in false-positive or false-negative values of PPV [16]. Patients urgently requiring a fluid bolus were excluded because they could not comply with the fluid management of our study procedure strictly.

Philips Intellivue MP60 monitors (Philips Medical Systems, Amsterdam, The Netherlands) were used for monitoring vital variables. Central venous catheters and thermistor-tipped arterial catheters in the femoral artery were inserted in subjects to connect a transpulmonary thermodilution device: PiCCO (Pulsion Medical Systems SE, Feldkirchen, Germany). Transpulmonary thermodilution variables such as global end-diastolic volume index (GEDI), cardiac index derived by pulse power analysis (CCI), PPV and cardiac index assessed by transpulmonary thermodilution (CI_**TPTD**_) were obtained from it. PiCCO device was adjusted by an engineer from manufacture according to the manufacturer’s protocol to ensure precision and accuracy of measurement every two weeks. All the ventilators available in our trial are Puritan Bennet 840. Assessment of no spontaneous breathing during ventilation was determined by respiratory flow signal analysis on the ventilator.

Before the measurement of PPV, all patients were titrated to maintain patients in a low/no-pain [Critical Care Pain Observation Tool (CPOT) score < 3] and moderately sedated [Richmond Agitation-Sedation Scale (RASS) score − 3] state [17, 18]. RASS is a 10-point scale, with four levels of anxiety or agitation (+ 1 to + 4 [combative]), one level to denote a calm and alert state (0), and 5 levels of sedation (− 1 to − 5) culminating in unarousable (− 5). A continuous infusion of propofol (0.3–2.0 mg/kg·h) was used to achieve a RASS score of -3 throughout the measurement. If this goal was not achieved, a continuous infusion of dexmedetomidine (0.2–1.2 μg/kg·h) and/or midazolam (0.04–0.15 mg/kg·h) was added. All patients were given remifentanil (2.0–5.0 μg/kg·h) for analgesia during measurement to maintain a CPOT score of < 3. The depth of sedation was modified and the spontaneous respiration was inhibited by adjusting the amount of drug infusion without using any muscle relaxant. The measurement started 1 h after the RASS and CPOT objective was reached and the cessation of spontaneous respiration was confirmed. All the patients achieved the goal of analgesia and sedation within the dosage range of drugs. Intravenous sedation was stopped when the patient reached a RASS score of –4 to –5. Intravenous analgesia was also stopped. Analgesics with sedatives were restarted at half the previous dose and adjusted accordingly to achieve the goals of sedation and analgesia.

**Fig. 1:**
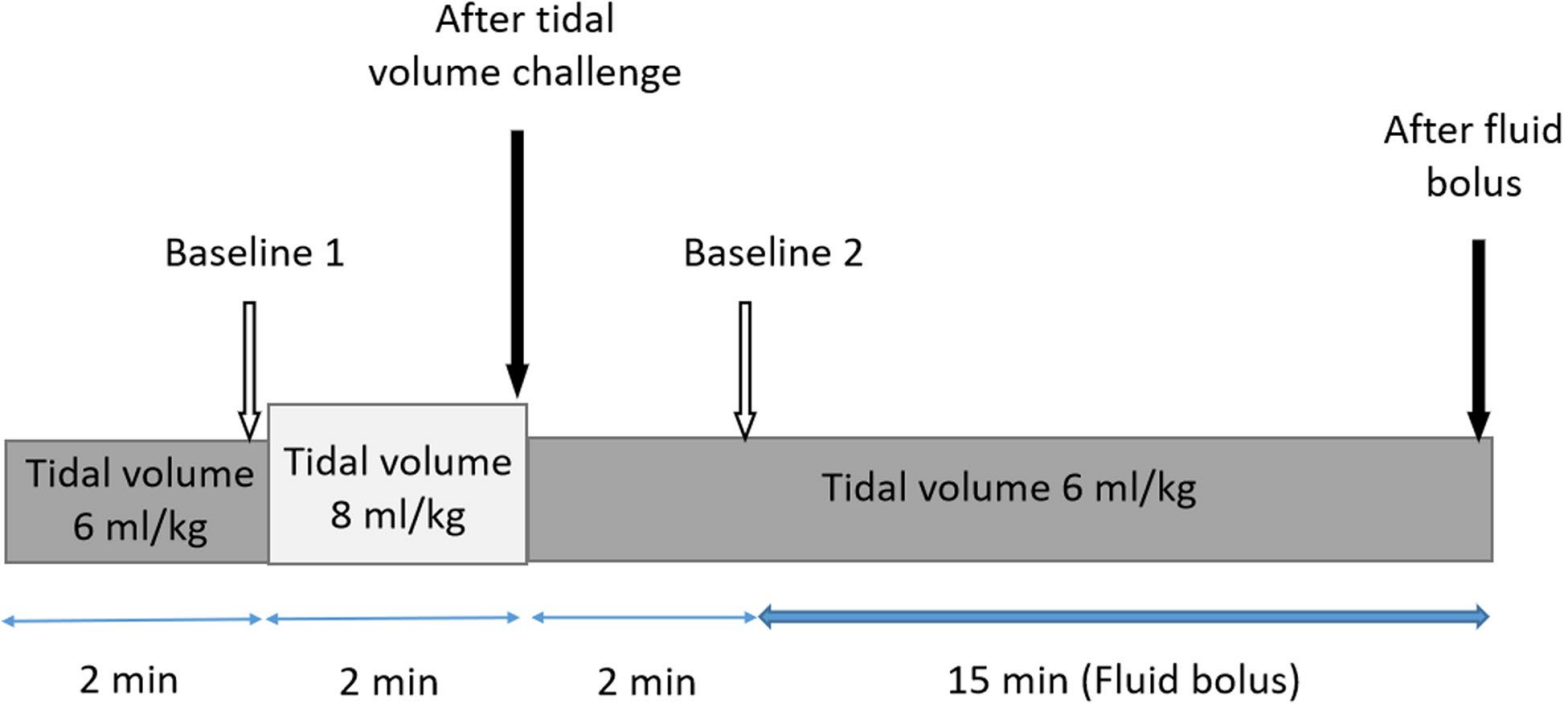
Study protocol. Arrows indicate time points at which measurements were made

The heart rate (HR), systolic blood pressure (SBP), diastolic blood pressure (DBP), mean arterial pressure (MAP), CCI, PPV, central venous pressure (CVP), plateau pressure (Pplat), positive end-expiratory pressure (PEEP), driving pressure (Pplat – PEEP), and static compliance of the respiratory system (Cst) were recorded at baseline and specific intervals (Fig. 1). All respiratory parameters were continuously monitored by the ventilator (Puritan Bennett 840 ventilator). Measurement of Cst was computed by dividing tidal volume by Pplat (measured during an end-inspiratory pause (2 s)) minus total PEEP.

Firstly, all subjects were ventilated in volume-controlled mode with a Vt 6 ml/kg predicted body weight (PBW) at baseline (baseline 1). Secondly, the "tidal volume challenge" was conducted by transiently increasing Vt to 8 mL/kg PBW for 2 min. Following this procedure, PPV_8_ (PPV at 8 ml/kg PBW) and other parameters were recorded during the 15 s before the end of the 2 min period. Then Vt was reduced back to 6 mL/kg PBW again for 2 min and relevant parameters were recorded (baseline 2). After that, 250 ml of crystalloid fluid bolus was given over 1*5* min, and measurements were repeated during the 1 min before the end of the 15 min period*.* Subjects *w*ere considered to be responders if there was an increase in the CI_TPTD_ of more than 15% after giving a fluid bolus at Vt 6 mL/kg PBW. Doses of vasoactive medications and PEEP were held constant. The change in PPV after transiently increasing tidal volume (ΔPPV_6-8_)was calculated.

### Statistical analysis

Measurement data conforming to a normal distribution were reported as mean ± SD; data that did not conform to a normal distribution are reported as the medians (25–75% interquartile range). Enumeration data were reported as frequency (percentage). The two groups of measurement data were compared with independent samples Student's t-test (normal distribution). The Wilcoxon rank-sum test was used for data that did not conform to a normal distribution, and the chi-squared test or Fisher's exact test was utilized to compare two groups of enumeration data. Paired sample t-test or a signed rank-sum test was used to compare paired data. An alpha value of *p* < 0.05 was considered to be significantly different. Receiver operating characteristic (ROC) curves of PPV and other parameters were plotted under different tidal volume values. The Youden method was used to identify the threshold values of PPV and other parameters and to identify optimal sensitivity and specificity for diagnosing the volume responsiveness of subjects [19]. The differences in the area under each ROC curve were compared using the Delong test [20]. The sample size estimation for a ROC analysis was based on research by Hanley et al. [21]. According to Xiaobo, the ratio of non-responders cases to responder cases was supposed to be about 0.7–1.4 [22]. Under the assumption of (1) type I error = 0.05 (2) type II error = 0.2 (3) acceptable discrimination was 0.7, the sample size should be at least 65 participants. The estimated sample size was corrected to 76 participants in consideration of at least a 15% drop-out rate. MedCalc version 20 (MedCalc Software Ltd, Ostend, Belgium) and SPSS version 24.0 software (USA) were used for all statistical analyses.

## Results

### Study population

**Fig. 2:**
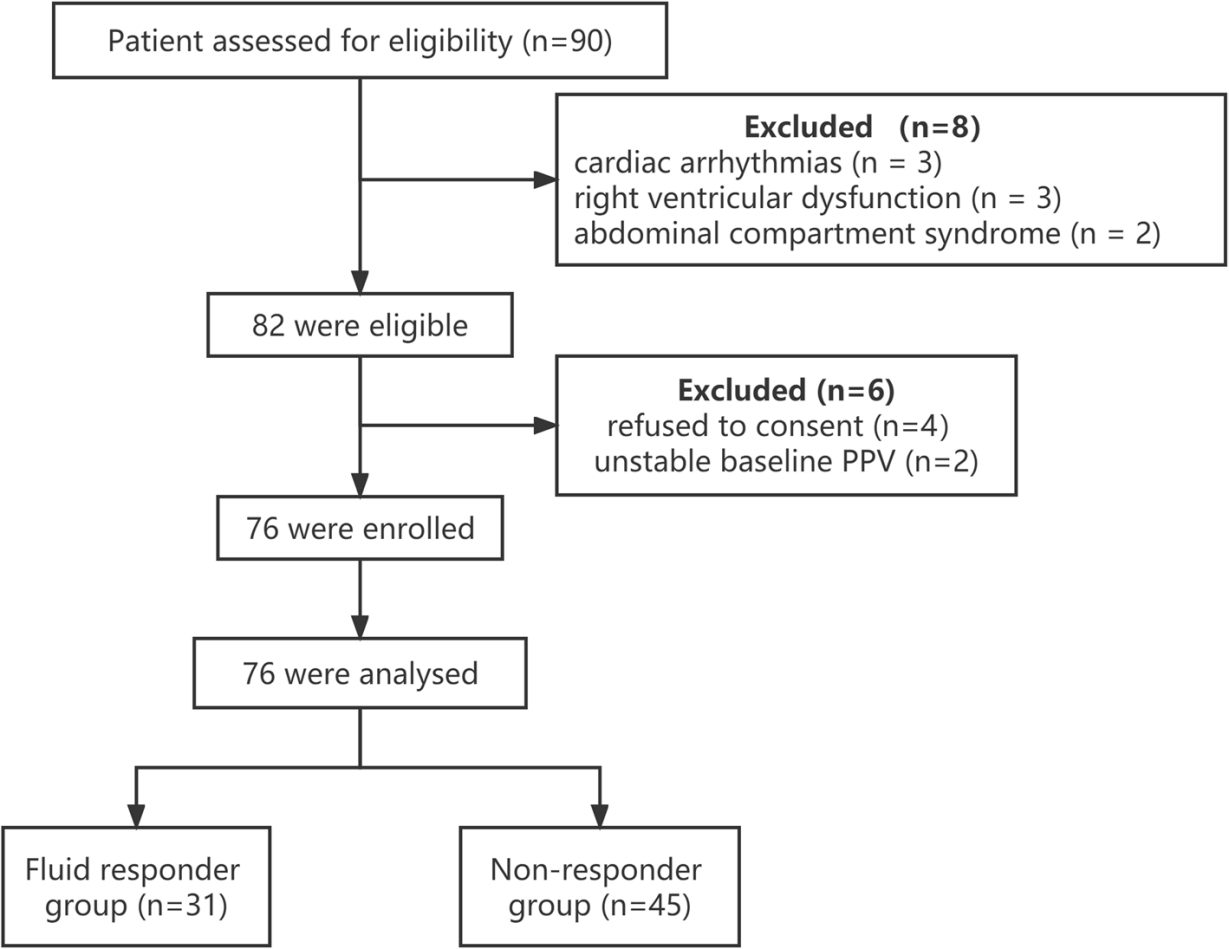
The flow of subjects in the study

In the enrollment period, 96 consecutive subjects were screened and 90 were considered eligible (cardiac output was not monitored in 4 subjects, acute circulatory failure in 2 subjects was reversed quickly). However, eight of the 90 subjects were excluded because of cardiac arrhythmias (*n* = 3), right ventricular dysfunction (*n* = 3), or abdominal compartment syndrome (*n* = 2). Four of the remaining guardians declined to sign informed consent. Data from 2 patients were excluded because of unstable baseline PPV. In the end, 76 subjects were analyzed (Fig. 2).

Fifty percent of the 76 subjects were men. The mean age of them was 55 ± 16 years. The mean Acute Physiology and Chronic Health Evaluation II score was 26 ± 9. All subjects had a diagnosis of acute circulatory failure and sepsis. Among them, 50 subjects had pulmonary infections, 13 subjects had intra-abdominal infections, 8 subjects had bacteremia, 5 had other sources of infection. The mean onset day of sepsis before inclusion in the current study was 5.7 ± 2.6 days. History of hypertension was present in 23% of subjects, diabetes mellitus in 9.2%, and dyslipidemia in 15.8%. Thirty-one of the 76 subjects (41%) were identified as responders because CO increased by greater than or equal to 15% after fluid challenge. Comparisons between responders and nonresponders are also shown in Table 1. For all of the above variables, there was no significant difference between responders and nonresponders. Before the fluid challenge, baseline hemodynamic and respiratory characteristics were compared between responders and nonresponders. Norepinephrine doses, heart rate, MAP, respiratory rate, tidal volume, PEEP, driving pressure, P_plat_, Cst, PaO_2_/FiO_2,_ and arterial lactate were not significantly different at baseline between fluid-responsive subjects and nonresponders (Table 2).

**Table 1:** Patients’ general characteristics at inclusion

Characteristics	Overall population (*n* = 76)	Fluid non-responders (*n* = 45)	Fluid Responders (*n* = 31)	*p*
Age (years)	55 ± 16	56 ± 16	53 ± 16	0.30
Male (%)	38(50%)	23 (51%)	15 (48%)	0.81
APACHE II score	26 ± 9	27 ± 10	24 ± 8	0.06
Onset time of sepsis (days)	5.7 ± 2.6	6.1 ± 2.4	5.1 ± 2.7	0.76
BMI	25 ± 4.4	25 ± 4.7	25 ± 3.9	0.60
Hypertension	18(23%)	11 (24%)	7 (23%)	0.85
Diabetes mellitus	7(9.2%)	4 (8.9%)	3 (9.6%)	0.91
Dyslipidaemia	12(15.8%)	7 (15.6%)	5 (16.1%)	0.94
Cause of infection				
Pulmonary	50(66%)	29 (64%)	21 (68%)	0.77
Abdominal	13(17%)	8 (18%)	5 (16%)	0.85
Blood stream	8(10.5%)	5 (11.1%)	3 (9.7%)	0.84
Others	5(6.6%)	3 (6.7%)	2 (6.4%)	0.97

Continuous data are presented as mean ± SD or median (range), as appropriate. Categorical data are presented as frequencies (percentage)

*APACHE II score* Acute Physiology and Chronic Health Evaluation II score, *BMI* Body mass index

**Table 2:** Baseline Hemodynamic and Respiratory Characteristics of Fluid Responders and Nonresponders

Parameters	Overall population (*n* = 76)	Fluid non-responders (*n* = 45)	Fluid responders (*n* = 31)	*p*
Norepinephrine dose (μg.kg^−1^.min^−1^)	0.55 ± 0.39	0.61 ± 0.41	0.45 ± 0.35	0.08
Heart rate (min^−1^)	112 ± 16	110 ± 18	116 ± 14	0.06
MAP (mm Hg)	76 ± 16	75 ± 16	78 ± 16	0.39
PPV (%)^a^	7.6 ± 3.1	6.7 ± 3.1	8.8 ± 2.7	0.004
CVP (mm Hg)	9.3 ± 2.3	9.8 ± 2.0	8.6 ± 2.5	0.025
CI_TPTD_ (L.min^−1^.m^−2^)	2.98 ± 0.53	2.88 ± 0.58	3.14 ± 0.40	0.003
GEDVI (ml.m^−2^)	783 ± 106	810 ± 88	743 ± 118	0.007
Respiratory rate (min^−1^)	27 ± 4	27 ± 4	27 ± 4	.807
Tidal volume (ml.kg^−1^ PBW)	6.0	6.0	6.0	.401
PEEP (cm H_2_O)	9.5 ± 2.8	9.9 ± 3.0	8.8 ± 2.4	.082
Pplat (cm H2O)	23 ± 3.3	23.1 ± 3.3	22.7 ± 3.4	.587
Driving pressure (cm H2O)	15 ± 2.0	15 ± 1.9	15 ± 2.0	.770
Cst (mL.cm H2O^−1^)	25.7 ± 3.8	26.1 ± 3.7	25.1 ± 3.9	.811
PaO_2_/FiO_2_ (mm Hg)	199 ± 71	193 ± 70	208 ± 73	.349
Arterial lactate (mmol.L^−1^)	3.8 ± 2.0	4.0 ± 2.1	3.4 ± 1.8	.193

Data are presented as mean ± SD or median (1st quartile to 3rd quartile)

P values were calculated between the fluid non-responders and fluid responders

*MAP* Mean arterial pressure, *PPV* Pulse pressure variation, *CVP* Central venous pressure, *CI*_*TPTD*_ Cardiac index assessed by transpulmonary thermodilution, *GEDVI* Global end-diastolic volume index, *PBW* Predicted body weight, *PEEP* Positive end-expiratory pressure, *Pplat* Plateau pressure of the respiratory system, *Cst* Static compliance of the respiratory system, *PaO*_*2*_ Partial pressure of arterial oxygen, *FiO*_*2*_ Inspired oxygen fraction

It was found that the CVP (8.6 ± 2.5 vs 9.8 ± 2.0, *p* = 0.025) and GEDVI (743 ± 118 vs 810 ± 88, *p* = 0.007) in the responders were lower than those in the nonresponders, while the PPV (8.8 ± 2.7 vs 6.7 ± 3.1, *p* = 0.004) and CI_TPTD_(3.20 ± 0.26 vs 2.83 ± 0.65, *p* = 0.003)in the responder group were higher than those found in the nonresponder group.

Influence of tidal volume challenge and fluid challenge in the responder and non-responder group.

The changes in hemodynamic parameters related to tidal volume challenge and fluid challenge are presented in Table 3. HR and MAP were not significantly different between the responder and nonresponder groups, regardless of whether before or after tidal volume challenge and fluid resuscitation. CCI and CI_TPTD_ seemed higher in the responder group than in the nonresponder group at all stages of our experiment. Driving pressure increased significantly in both groups (15 ± 1.9 to 20 ± 2.2 in non-responders, 15 ± 2.0 to 20 ± 2.5 in responders) at the end of the Vt challenge while Cst remained the same. Before the fluid challenge, CVP, PPV, and GEDVI were recorded when a tidal volume of 6 ml/kg ventilation was performed (baseline 1). It was found that PPV (6.8 ± 3.1 vs 8.8 ± 2.7), CCI (2.88 ± 0.58 vs 3.14 ± 0.40), and CI_TPTD_ (2.89 ± 0.58 vs 3.16 ± 0.36) in the nonresponder group was lower than in the responder group, and the CVP (9.8 ± 2.0 vs 8.6 ± 2.5) and GEDVI (810 ± 88 vs 743 ± 119) in the nonresponder group were higher than those found in the responder group.

**Table 3:** Evolution of hemodynamic Variables in fluid responders and nonresponders at baseline or after fluid challenge

	non responder (*n* = 45)				responder (*n* = 31)			
	Baseline 1	After Vt Challenge	Baseline 2	After Fluid Challenge	Baseline 1	After Vt Challenge	Baseline 2	After Fluid Challenge
	Vt, 6 mL/kg	Vt, 8 mL/kg	Vt, 6 mL/kg	Vt, 6 mL/kg	Vt, 6 mL/kg	Vt, 8 mL/kg	Vt, 6 mL/kg	Vt, 6 mL/kg
Heart Rate	110 ± 18	109 ± 18	109 ± 18	107 ± 17	116 ± 14	116 ± 14	116 ± 14	112 ± 14
MAP (mm Hg)	75 ± 16	76 ± 14	77 ± 14	76 ± 15	78 ± 16	74 ± 18	74 ± 18	82 ± 15
CCI (L/min/m^2^)	2.88 ± 0.58	2.80 ± 0.77	2.81 ± 0.60	2.95 ± 0.62	3.14 ± 0.40^a^	3.20 ± 0.56^a^	3.18 ± 0.32^a^	3.71 ± 0.44^ac^
CVP (mm Hg)	9.8 ± 2.0	11.1 ± 2.3	10.0 ± 2.1	13.3 ± 3.4^c^	8.6 ± 2.5^a^	9.9 ± 2.4^a^	8.5 ± 2.6^a^	10.4 ± 2.5^ac^
PPV (%)	6.8 ± 3.1	8.5 ± 3.0^b^	6.7 ± 3.5	5.4 ± 4.6	8.8 ± 2.7^a^	13.0 ± 1.7^ab^	8.7 ± 3.2^a^	6.2 ± 3.8^c^
CVP_6-8_		1.5 ± 1.3				1.4 ± 1.1		
PPV_6-8_		1.7 ± 1.1				4.2 ± 1.6^a^		
Driving pressure (cm H_2_O)	15 ± 1.9	20 ± 2.2^b^	–	–	15 ± 2.0	20 ± 2.5^b^	–	–
Cst (mL.cm H_2_O^−1^)	26.1 ± 3.7	26.3 ± 3.3	–	–	25.1 ± 3.9	25.2 ± 3.6	–	–
CI_TPTD_ (L.min^−1^.m^−2^)	2.89 ± 0.58	–	–	2.93 ± 0.57	3.16 ± 0.36^a^	–	–	3.74 ± 0.37^ad^
GEDVI (mL.m^−2^)	810 ± 88	–	–	806 ± 136	743 ± 119^a^	–	–	752 ± 120

*Vt* tidal volume, *MAP* Mean arterial pressure, *CCI* Continuous cardiac index which was provided by pulse contour analysis, *CI*_*TPTD*_ Cardiac index assessed by transpulmonary thermodilution, *Cst* static compliance of the respiratory system, *GEDVI* Global end-diastolic volume index

Values are expressed as mean ± sd

^a^*p* < 0.05, fluid responders vs fluid nonresponders

^b^*p* < 0.05, Vt 8 mL/kg vs baseline 1 (Vt = 6 ml/kg)

^c^*p* < 0.05, baseline 2 vs after fluid bolus

^d^*p* < 0.05, baseline 1 (Vt, 6 mL/kg) vs after fluid bolus (Vt, 6 mL/kg PBW)

Dashes indicate variables that were not measured in monitoring CI_TPTD_ and GEDVI

When 8 mL/kg Vt ventilation was performed, it was observed that CVP (11.1 ± 2.3 vs 9.9 ± 2.4) remained higher while PPV (8.5 ± 3.0 vs 13.0 ± 1.7) was still lower in the nonresponder group than in the responder group. PPV increased in both groups. However, the change extent of PPV in the responder group was more significant (4.2 ± 1.6 vs 1.7 ± 1.1). The changing size of CVP showed no difference between the responder group and the nonresponder group. At baseline 2, all hemodynamic parameters in both groups were similar to those measured at baseline 1 (baseline 1 vs baseline 2). After the fluid challenge, CVP increased in both groups while PPV decreased in responder groups. Meanwhile, no significant difference could be noted in GEDVI in both groups.

### ROC curve analysis

**Fig. 3:**
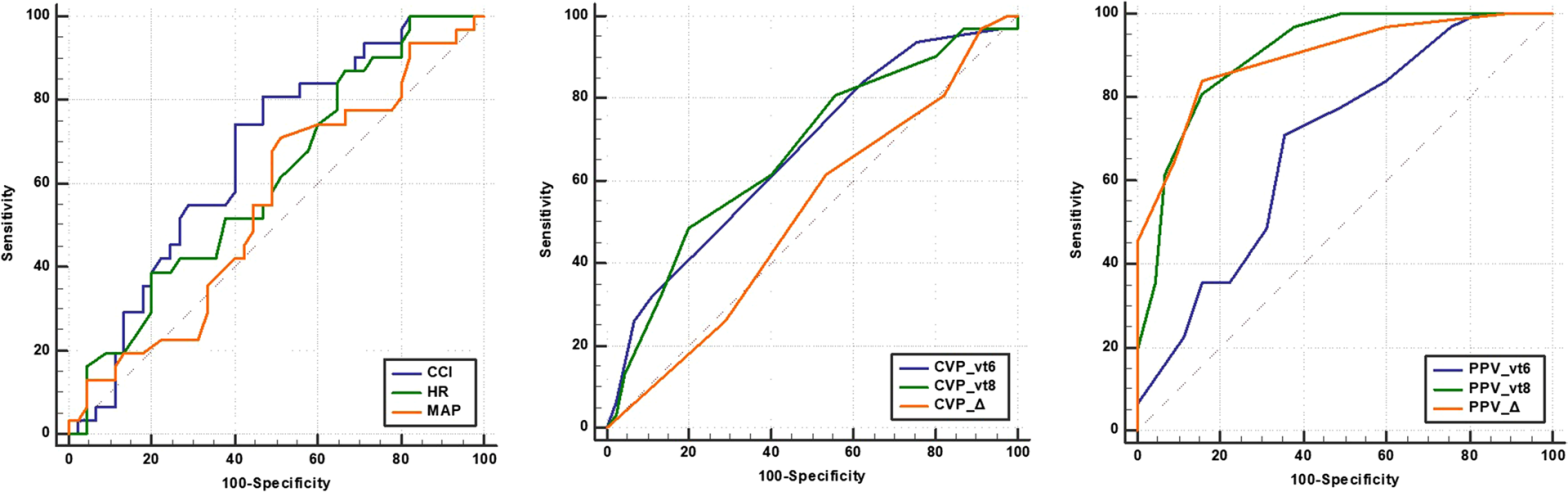
Receiver operating characteristics curves from nine diagnostics tests to detect fluid responsiveness. CCI, continuous cardiac index during ventilation with 6 ml/kg predicted body weight tidal volume; HR, heart rate; MAP, mean arterial pressure; CVP_vt6, central venous pressure during ventilation with 6 ml/kg predicted body weight tidal volume; CVP_vt8, central venous pressure during ventilation with 8 ml/kg predicted body weight tidal volume; CVP_Δ, changes in central venous pressure between ventilation with 6 and 8 ml/kg predicted body weight tidal volume; PPV_vt6, pulse pressure variation during ventilation with 6 ml/kg predicted body weight tidal volume; PPV_vt8, pulse pressure variation during ventilation with 8 ml/kg predicted body weight tidal volume; PPV_Δ, change in pulse pressure variation between ventilation with 6 and 8 ml/kg predicted body weight tidal volume

In the ROC curve analysis, MAP and heart rate showed no predictability for fluid responsiveness. The predictive power of PPV_6_ was limited with an AUC of 0.69 (95% CI 0.57–0.79,*p* = 0.002). While PPV_8_ significantly improved predictive ability with an AUC of 0.90 (95% CI 0.81–0.96, *p* < 0.001). The predictive power of ΔPPV_6–8_ was similar to PPV_8_ with an AUC of 0.90 (95% CI 0.80–0.95, *p* < 0.001). CCI, CVP_6_, CVP_8_ and ΔCVP_6–8_ showed weak predictability for fluid responsiveness with AUCs of 0.67 (95% CI 0.55–0.72, *p* = 0.007), 0.67 (95% CI 0.55–0.75, *p* = 0.007), 0.68 (95% CI 0.56–0.78, *p* = 0.008) and 0.52 (95% CI 0.40–0.53, *p* = 0.81) respectively. The optimal threshold values of PPV_8_ and ∆PPV_6–8_ were 11% (sensitivity 80%, specificity 84%) and 2% (sensitivity 84%, specificity 84%) respectively (Table 4 and Fig. 3).

**Table 4:** Diasgnostic ability of different parameters to predict fluid responsiveness

	AUC(95%CI)	*p*	cut off value	Youden index	Sensitivity (%)	Specifificity (%)
MAP	0.55(0.43–0.66)	0.466	72	.198	71	49
Heart rate	0.61(0.48–0.71)	0.102	99	.203	87	33
CCI	0.67(0.55–0.72)	0.007	2.96	0.342	74	60
PPV_6_	0.69(0.57–0.79)	0.002	7	.354	71	64
PPV_8_	0.90(0.81–0.96)	< 0.001	11	.651	80	84
ΔPPV_6–8_	0.90(0.80–0.95)	< 0.001	2	.683	84	84
CVP_6_	0.67(0.55–0.77)	0.007	10	.216	84	38
CVP_8_	0.68(0.56–0.78)	0.008	9	.284	48	80
ΔCVP_6–8_	0.52(0.40–0.63)	0.81	1	.079	61	47

Receiver-operating characteristic curves comparing the ability of various variables to discriminate between fluid responders and nonresponders

*AUC* Area under the receiver operating characteristic curve, *CI* Confidence interval, *MAP* Mean arterial pressure, *CCI* Continuous cardiac index, *Vt* tidal volume, *PPV*_*6*_ PPV at Vt 6 mL/kg PBW, *PPV*_*8*_ PPV at Vt 8 mL/kg PBW, *CVP*_*6*_ CVP at 6 mL/kg PBW, *CVP*_*8*_ CVP at 8 mL/kg PBW, *ΔPPV*_*6–8*_ Change in PPV after increasing Vt from 6 to 8 mL/kg PBW, *ΔCVP*_*6–8*_ Change in CVP after increasing Vt from 6 to 8 mL/kg PBW, *PBW* Predicted body weight, *PPV* Pulse pressure variation

## Discussion

This study demonstrated that PPV was not reliable in predicting fluid responsiveness in our subjects under protective ventilation. However, the predictive ability could be enhanced by increasing the tidal volume from 6 ml/kg to 8 ml/kg temporarily. The tidal volume challenge did not improve the predictive power of CVP. Absolute change in PPV values obtained by the Vt challenge (ΔPPV_6–8_) can predict fluid responsiveness with similar predictive capability compared to PPV_8_. The optimal threshold of ∆PPV_6–8_ was > 2%.

These findings could be expected as cardiopulmonary interactions, which is the underlying mechanism behind PPV, are highly correlated with the extent of airway pressure transmission to intrapleural pressure [8]. This transmission is inversely related to the elastance of the lung and chest wall [23], while there is a linear correlation between it and the ratio of the chest wall to respiratory system elastance [11]. It was suggested that detection of PPV would have a high false-negative rate in patients with ARDS under protective ventilation accounting for low Vt and low respiratory system compliance in them.

It had been demonstrated that the use of relatively low Vt in patients of ARDS could reduce mortality. However, subsequent data analysis supported the benefit of using lower VT in patients without a diagnosis of ARDS. One example was the protective effect of low Vt on the development of ARDS and/or pneumonia [24]. It was also suggested efforts be made to achieve low tidal volume ventilation in all patients with lung injury or undergoing mechanical ventilation for some reason [25]. So we also included subjects receiving low tidal ventilation without ARDS. Accuracy measurements of PPV required the shortage of spontaneous respiratory effort as it would interfere with the controlled, cyclic variation in intrathoracic pressure [16]. So we inhibit spontaneous respiratory effort during the measurement of PPV, not in the whole period of treatment.

Positive pressure ventilation could result in cyclical changes in intrathoracic pressure, which induce variations in stroke volume. That is how PPV generates. PPV would be inaccuracy if the cyclic changes in intrathoracic and transpulmonary pressures are not large enough to affect preload, which might be too small when subjects were ventilated with low tidal volumes [26]. The heart–lung interactions, as well as the intrathoracic pressure, could be amplified by increasing tidal volume from 6 to 8 mL/kg. Fluid responsiveness could be recognized in these settings. Therefore, the “tidal volume challenge” helped identify fluid responders as PPV increased significantly only in responders [27].

Our study showed that performing a tidal volume challenge mildly enhanced the reliability of the PPV test. Similar to our results, another study developed by Myatra confirmed that change in PPV after a "Vt challenge" reliably predicted fluid responsiveness [27]. However, the AUC of PPV_8_ and ΔPPV_6–8_ that we found (0.90 and 0.90 respectively) seemed a little lower than those reported in that study (0.91 and 0.99, respectively). One of the possible reasons for these results was that subjects enrolled in our study had lower static compliance of the respiratory system than those enrolled in that study ( 26 ± 4 vs 29 ± 8). As it had been suggested, pulse pressure variation became less accurate for predicting fluid responsiveness when the compliance of the respiratory system was ≤ 30 ml/cm H_2_O [28].

Our study also suggested that baseline central venous pressure did not accurately predict fluid responsiveness. This finding was consistent with the results of many other studies [7, 29]. However, surveys regularly report that CVP was still used for predicting fluid responsiveness by many clinicians [30, 31]. Although CVP could not reflect volume responsiveness, it does not mean that CVP should not be measured in subjects with or at risk of acute circulatory failure as the CVP is a good marker of preload (not preload responsiveness) and a key determinant of cardiac function and the pressure gradient for organ perfusion [32].

Another interesting finding in our study was that CCI in the responder group was higher than in the non-responder group (3.14 ± 0.40 vs 2.88 ± 0.58), which appeared to contribute to fluid responsiveness prediction mildly (AUC 0.67). The possible mechanism behind this observation was that a subject's response to fluids depends on both preload and cardiac contractility. Fluid responsiveness could only be predicted accurately in cases with normal ventricular contractility [33]. We supposed the decreased CCI in the non-responder group might derive from a reduced force of ventricular contraction, the circumstance under which fluid responsiveness could not be predicted accurately with an increase of preload.

The present study had the following limitations. First, we did not measure fluctuation of intrapleural pressure. It was pointed out that PPV adjusted by respiratory variations in pleural pressure could improve the prediction of fluid responsiveness [11]. However, measuring intrapleural pressure has faced challenges in implementation in real-world settings accounting for the complexity of this technology. Second, the study population consisted of only a small number of highly selected subjects with severe sepsis. Our results require validation in a larger and more heterogeneous population. Third, we did not record the volume of fluid received before inclusion not only because the volume of feed and fluid administered was not documented accurately but also because our study only tried to detect whether the "tidal volume challenge" could help predict fluid responsiveness and identify true responders at any volume status.

## Conclusion

The change in PPV following the Vt challenge test has good but not exceptional reliability in predicting fluid responsiveness in subjects with low respiratory system compliance employing small tidal volume ventilation.

## Supplementary Information

**Additional file 1.**

## Data Availability

The datasets used during the current study are available as a supplementary file.
